# Multiple phonological activation in writing: evidence for cascadedness in Chinese written verb production

**DOI:** 10.3389/fpsyg.2024.1330522

**Published:** 2024-01-30

**Authors:** Xuebing Zhu

**Affiliations:** Institute of Linguistics, Shanghai International Studies University, Shanghai, China

**Keywords:** word production, handwriting, verb, phonology, Chinese

## Abstract

The dynamics of information transmission through the lexical system during written word production remain underspecified. Existing studies largely come from noun production, relatively less work has explored verb production. Verbs, representing actions or states, are considered more abstract and are found to be more challenging to be produced. The present study investigated phonological involvement and the principles governing information flow during Chinese written verb production. Participants wrote down single verbs and verb phrases while ignoring phonologically related, or unrelated distractor pictures. Results revealed phonological facilitation effects on writing latencies from phonologically related distractors in the verb phrase generation. Findings provide novel chronometric evidence that information transmission during written production involves cascaded activation allowing multiple phonological codes to be activated prior to written output. This phonological facilitation effect signifies the influence of phonology, especially lexical phonology, has been underestimated in writing.

## Introduction

A central question in word production concerns how information flows between semantic, phonological and orthographic levels during the process of conveying a preverbal message into spoken or written output (Dell, 1986; Caramazza and Hillis, 1990; Van Galen, 1991; Rapp et al., 1997; Levelt et al., 1999). Models of word production generally propose that following conceptual preparation, lexical items corresponding to the preverbal message are selected at the lemma level. This is followed by retrieval of word form information, including phonological codes, and orthographic codes specifying written form of the word. To some extent, the processing levels in handwriting have been identified are similar to those involved in spoken-word production (Bonin et al., 1998; Bonin and Fayol, 2000). Nevertheless, handwriting is not merely a byproduct of speech but possesses distinct characteristics different from spoken language production.

Information transmission has been rigorously examined by whether the extraction of lexico-semantic and word-form processing unfolds in a sequential manner or if a level of interaction exists between the two elements (for a comprehensive review, refer to Levelt et al., 1999). In spoken output, this issue has been extensively investigated with enormous typical tasks and still in a mixture of findings on discrete-serial or cascaded (and interactive) way of information transmission. That is, a central question concerns on whether phonological encoding is restricted only to a selected lexical node (discrete-serial, Levelt et al., 1999), or whether multiple candidates at the semantic level activate their phonological representations in a cascaded manner (Humphreys et al., 1988; Morsella and Miozzo, 2002) or an interactive way, with an additional assumption that bidirectional transmission of activation between semantic and phonological encoding (Dell, 1986). However, much is unknown, and there is a lack of empirical evidence on how information is transmitted from semantics to phonology in written production. A major reason is the role of phonology in written production. Firstly, it must be established whether phonology is involved, before examining how it influences writing. To a large extent, existing research on the role of phonology has focused on the interplay between phonology and orthography in information transmission. It is important to first review this evidence.

A long-standing debate in the literature concerns whether phonological information constrains orthographic processing: whether access to orthographic codes depends obligatorily on prior phonological encoding, or whether orthographic codes can be accessed directly from the semantic system without phonological mediation.

## Models of phonological processing route during written production

According to the phonological mediation hypothesis (Geschwind, 1969), phonological codes are activated prior to and constrain orthographic output. This view was supported on observations that individuals engage in subvocal articulation during writing, and that phonology-based errors sometimes arise in written production (e.g., *their - there*; Aitchison and Todd, 1982). Further evidence comes from neuropsychological patients with comparable impairments in speaking and writing, suggesting a shared reliance on phonological codes (for reviews see Basso et al., 1978).

In contrast, the orthographic autonomy hypothesis proposes that lexical orthographic representations can be accessed directly from the semantic system without obligatory phonological mediation (Miceli et al., 1997; Rapp et al., 1997). This view is supported by dissociations between spoken and written production in neurological patients, such as those who can write words they cannot say correctly, without a deficit in the semantic system (Bub and Kertesz, 1982; for reviews see Tainturier and Rapp, 2001). The existence of such selective impairments in either speech or writing has been taken as evidence that orthographic lexical entries can be independently accessed.

Of critical interest was whether onsetting phonological effects preceded orthographic effects as predicted by obligatory phonological mediation, or if orthographic effects emerged earlier consistent with direct orthography-semantic mappings. Reaction time measures provide limited temporal granularity regarding component dynamics. In contrast, event-related potentials (ERPs) enable direct millisecond-resolution recording of neural activity time-locked to stimuli, furnishing much finer-grained chronometric evidence adjudicating phonological mediation and orthographic autonomy.

Combined with factorial manipulations, ERPs can sensitively index the relative timecourse of effects linked to representational codes, as exploited by Zhang and Wang (2016). Using a written picture-word interference paradigm, ERPs were measured during presenting distractors orthographically, phonologically, or both orthographically and phonologically related to picture names. Critically, orthographic effects on the N400 emerged substantially earlier, from 370−460 ms, than phonological effects which arose later from 460−500 ms after picture onset. This timing clearly demonstrates initial direct semantic-orthography mapping. The later phonological effect likely reflected activation spreading from orthography to phonology rather than initial feedforward transmission as argued by phonological mediation. These neural dynamics provide compelling temporal evidence against obligatory phonological mediation in Chinese writing production (in line with Zhang and Wang, 2015; but see Qu and Damian, 2020).

Whether phonological codes influence written production, and the time course of phonological involvement relative to orthographic access if present, has major consequences for models of writing. Notably, it is pivotal to acknowledge the role of phonology in written production, even from the perspective of the orthographic autonomy hypothesis (Zhang and Wang, 2016). Bonin and Fayol (2000) firstly presented empirical evidence on the role of phonology during written production in neurologically healthy participants. Alongside with the consensus of directly mapping of semantic-to-orthography, their model of dual-route phonological meditation additionally provides flexibility in generating orthographic outputs from phonological inputs at both lexical and sublexical levels. The lexical route involves spreading activation from semantic representations to phonological lexical entries, which then pass activation to corresponding orthographic lexical entries. The sublexical route involves a sublexical phonology to orthography conversion (POC), that activate graphemes corresponding to the phonemes of the phonological word (Rapp and Caramazza, 1997; Bonin and Fayol, 2000). Converging evidence comes from a fine-grained time course of lexical phonological activation [in 202−264 ms, e.g., target picture: “海洋” (/hai3yang2/, ocean)-distractor “孩子” (/hai2zi/, child)] and sublexical processing [“海洋” (/hai3yang2/, ocean)-distractor “妹妹” (/mei4mei4/, sister) in 370−470 ms] in Chinese written production (Wang and Zhang, 2022).

As can be seen, when it comes to the role of phonology, it contributes to writing, starting from semantic-to-lexical phonology mapping. The investigation into the nature of activation flow, whether it is serial or cascaded, during the act of writing has been scarcely addressed (Bonin and Fayol, 2000). This paucity of research is, in part, attributable to the inherent complexities associated with alphabetic languages like English, where orthography and phonology are intricately intertwined, complicating the design of studies aimed at isolating these variables. Unlikely, non-alphabetic scripts, such as Chinese, present a unique opportunity for investigation (Qu et al., 2011; Zhu et al., 2015; Wang and Zhang, 2022). The distinct separation between orthography and phonology in these languages facilitates a more nuanced examination of their respective roles and interactions. The present study is situated within this context, aiming to elucidate the multiple phonological activation in the context of Chinese written production. A central question guiding this inquiry is whether lexical access unfolds in a cascaded or serial fashion during the writing process.

## Discrete-serial verus cascadedness in spoken and written word production

Turning to the examination of potential multiple phonological activation, the discrete two-stage theory posits that only the phonological information of the target item is activated, negating the activation of non-target phonological information. Levelt et al. (1991) delved into this by employing a lexical decision task with auditory probe words. Participants were primarily tasked with naming pictures. Sequentially, pictures were presented on a screen, and in certain instances, an auditory probe word would follow, preceding the naming response to the picture. In these scenarios, participants were required to make a lexical judgment (word or non-word) on the auditory probe word. The relationship between the auditory probe word and the picture could be semantically, phonologically, or semantically-mediated phonologically related. For instance, for a picture of a “sheep,” the auditory probe could be “goat” (semantically related), “sheet” (phonologically related), or “goal” (phonologically related through the semantic intermediary “goat”). The results did not detect phonological activation for semantically-mediated items, aligning with the discrete two-stage theory’s predictions: only the target item’s phonological information is processed, with no evidence of non-target multiple phonological activation, suggesting a discrete processing. Similarly, Jescheniak et al. (2003), employing the more sensitive Electroencephalography technique, failed to detect non-target phonological activation in German. From another point of view, in an earlier stage of language production field, a study of Schriefers et al. (1990), the Picture-Word Interference (PWI) paradigm was employed to scrutinize naming response times. Their findings revealed that at a Stimulus Onset Asynchrony (SOA) of −150 ms, there was evidence of semantic activation, yet phonological activation was conspicuously absent. Conversely, at SOAs of 0 and 150 ms, phonological activation was evident, while semantic activation was not observed. These results lend robust support to the perspective that semantic and phonological activation proceed in a strictly sequential and independent manner. Collectively, these studies bolster the notion that within the language production system, semantic and phonological processing transmitted in a serial manner, with lexical selection and phonological encoding being distinctly separate stages.

In contrast, interactive and/or cascaded models propose that multiple lexical nodes activated on the basis of the preverbal message transmit activation concurrently to orthography and phonology (Dell, 1986). Introducing the Picture-Picture Interference (PPI) paradigm, which turns to be one of the most influential tasks, Morsella and Miozzo (2002) investigated non-target phonological activation in spoken language production. In this paradigm, a distractor picture was superimposed on the target picture. Participants were instructed to name the green-colored picture (the target) while ignoring the red-colored picture (the distractor). The distractor pictures were either phonologically related to the target picture names (e.g., target picture name “bed” with distractor picture name “bell”) or phonologically unrelated (distractor picture name “hat”). The findings revealed that naming of the target picture was significantly faster in phonologically related condition compared with unrelated condition. This suggests that the phonological information of the distractor picture name was activated during the production of the target picture name, indicating the presence of multiple phonological activation during spoken word production. Furthermore, Meyer and Damian (2007) established varying degrees of phonological relatedness between the target and distractor pictures (homophones, onset related, rhyme related, and unrelated conditions) and consistently observed phonological facilitation effects.

Nevertheless, researchers have argued a possibility that this phonological facilitation effect might stem from the mis-selection of the target picture and the context picture, which are superimposed at the screen’s center (Navarrete and Costa, 2005; Roelofs, 2008). However, a growing number of evidence from color-picture naming (Kuipers and La Heij, 2009) and a word association task demonstrated the validity of phonological facilitation effect, when the target was physically different from the context picture (Humphreys et al., 2010; but see null effects in Chinese, Zhang and Zhu, 2016). Thus, this phonological facilitation effect could be considered as solid index of multiple phonological activation. These studies underscore the notion that the phonological nodes of non-target items are indeed activated, supporting the cascaded model.

When it comes to the relatively uncharted territory of written production, the sparse yet critical studies have provided interesting findings. To elucidate the relationship between semantic and form-related processing stages, researchers factorially crossed semantic and phonological/orthographic relatedness in picture-word interference paradigms. The prevalent finding is an attenuation of the semantic interference effect with mixed distractors that are both semantically and phonological/orthographic related to targets. Namely, pairs like “*rabbit”* (a target picture) – *“rat”* (a mix distractor) predominantly function as form-related, with the anticipated semantic effect from shared category membership being considerably subdued (e.g., Starreveld and La Heij, 1995, 1996; Damian and Martin, 1999). More importantly, this typical finding is consistent not only in spoken output as outlined above, but also in written production (Bonin and Fayol, 2000). As can be seen, this robust finding has been taken as evidence for interactive and/or cascaded models constrain mappings from semantic to phonological/orthographic related processing.

The intricate relationship between phonology and orthography in alphabetic languages has historically rendered their separation a challenging endeavor. This inherent complexity has catalyzed a growing wave of scholarly inquiry aimed at delineating these intertwined elements. In response to this challenge, Roux and Bonin (2012) systematically manipulated the orthographic and phonological overlap between target and context pictures presented in a PPI paradigm to provide valuable insights into how information cascades through the lexical system during written naming. Writing latencies were shorter in the both orthographically and phonologically related versus unrelated condition, extending previous spoken naming findings to the written modality as outlined above.

However, with maximumly isolating potential orthographic effects and phonological effects, in French target and context pictures shared the initial letter but not the initial sound, as in “cigar–camion,” or they shared the initial phonemes but not the initial letter, as in “singe–ceinture,” only the orthographically related condition facilitated written latencies, rather than the phonologically related condition. Consistently, using the same experimental paradigm, Qu and Damian (2015) has found that orthographic facilitation effects exist even in a non-alphabetic language (Chinese) where phonology and orthography are largely dissociated. This semantic-to-orthography cascading effect, provides strong evidence that cascading originates from semantic activation of multiple lexical nodes directly to their orthographic forms.

Upon closer examination of those studies, which offer a comprehensive backdrop for the present research, only one study on the mapping between semantic and pure phonological processing in writing fails to find evidence for activation of multiple phonological candidates (Roux and Bonin, 2012). As writing relies on accessing orthographic rather than phonological codes, it is important to investigate whether principles established for speech generalize to the written modality. The present study, detailed subsequently, is designed to contribute to our understanding of the intricate processes underpinning written word production.

## The present study

The present study aims to use non-alphabetic languages (Chinese) to examine whether multiple phonological activation manifest differently: firstly, how distinct phonological structures might influence the multiple phonological activation. Existing studies elucidate that the primary phonological units differ across languages: while alphabetic languages like English predominantly operate with phonemes as their primary phonological units, non-alphabetic languages such as Chinese primarily utilize syllables (O’Seaghdha et al., 2010). Secondly, in Chinese, phonology and orthography are largely dissociated. Chinese characters correspond to syllables with clear syllable boundaries, and there is little resyllabification, allowing direct examination of mappings from semantic to lexical-phonology (syllables, Zhu et al., 2015), without the involvement of compounding from orthography. Those discrepancies underscore the profound influence of linguistic structure on phonological processing and highlight the necessity of considering language-specific phonological units when investigating language production.

The current study attempts to extend the word association and picture-picture interference paradigms by taking advantage of the facilitative properties of verbs in both paradigms. Participants were presented with a probe word and distractor picture simultaneously, with instructions to ignore the picture and write down the single corresponding verb relative to the presented probe word as quickly as possible (e.g., “*Watch* TV,” “*TV”* as a probe to generate target verb *“watch”*). Two different types of verb context were designed to generate the single verb and verb phrase. To circumvent the direct presentation of probe words in the verb phrase task, an innovative approach akin to picture-picture interference paradigm is employed. Instead of presenting probes (*“TV”*) directly, participants are exposed to green probe pictures and are instructed to write down the complete verb phrase (“*watch TV”*) while ignoring red distractor pictures. In phonologically related trials, the names of the accompanying pictures were phonologically similar to the target verb response (“wall-*watch”*). In unrelated trials, probe words and pictures were rearranged so that there was no phonological similarity to the target (“duck-*watch”*). This methodology facilitates an intricate examination of the activation flow from semantic to lexical-phonology in Chinese verb production, without the direct presentation of textual probes.

The incorporation of verbs in this study is twofold: it emanates from an enhancement in the experimental paradigm and is rooted in the intricate processing dynamics of verbs. As fundamental linguistic categories, the processing and retrieval of verbs are inherently more time-consuming and complex compared to nouns (Vigliocco et al., 2002). Unlike the extensive studies focusing on noun objects, verb research remains relatively uncharted.

A wealth of neuropsychological evidence corroborates the distinct cerebral representations and processing mechanisms for nouns (the left temporal cortex) and verbs (the left posterior frontal areas, Breining et al., 2021). Naming deficits, exhibiting a double dissociation in primary progressive aphasia, are underscored by the pronounced impairments in verb naming in non-fluent and logopenic variants, contrasted by significant noun naming deficits in the semantic variant (Thompson et al., 2012). These distinctions are potentially attributable to the disparate semantic and grammatical properties inherent to nouns and verbs. During lexical access, the extraction of semantic and grammatical information is pivotal. The degrees of activation of these informational facets influences the activation at the phonological level. Although both nouns and verbs activate semantic and grammatical information during lexical selection, the complexity of verb semantics and the concurrent activation of grammatical information render verbs more activated at the lexical selection level (Vigliocco et al., 2006). The study of verbs is not only intriguing but also essential for understanding language production. The intricate processes involved in verb usage offer insights into the complex interplay of cognitive, neural, and linguistic mechanisms that underpin effective communication. Unraveling the nuances of verb processing can illuminate the multifaceted nature of language production and comprehension, contributing to enhanced pedagogical strategies and interventions for language disorders.

## Materials and methods

### Participants

Twenty-four students (5 male; average age 21.4 years; range 18−31 years) for single verb production and thirty students (9 male; average age 22.5 years; range 18−29 years) for verb phrase production from Shanghai International Studies University were paid for their participation. All were native Chinese speakers and had normal or corrected-to-normal vision.

### Materials and design

Thirty-six-line drawings of common objects were selected from Zhang and Yang (2003)’s picture database with a few specifically prepared for purposes (Vigliocco et al., 2002). All pictures had disyllabic names. Twenty-four probe words were selected. In the single verb generation, participants were asked to generate the first verb (response) corresponding to the probe word. The experiment manipulated the phonological relationship between picture and response verb word. These words shared syllables with the first character of context picture names [i.e., a target verb 看 (/kan4/, “watch”), 电视 (/dian4shi4/, “TV”) as a probe word, 砍刀 (/kan3dao1/, “chopper”) as a context picture]. In the verb phrase generation task, the probe word was replaced by the corresponding probe picture by ruling out the direct presentation of visual word form. Participants were asked to ignore the context picture in red line, but write the first verb phrase corresponding to the probe picture in green line. All 24 probe-response-picture stimuli sets were used in the phonologically related condition. The same response words and pictures were then recombined to form phonologically unrelated conditions.

The degree of match between the target verb and the probe word was assessed before the study. Using a 5-point scale, 20 linguistics students from the same participant pool (2 males, aged 18−27 years) were asked to rate the degree of verb-probe match based on their linguistic knowledge. Scores from 1 to 5 indicated that the degree of was very inappropriate, inappropriate, uncertain, appropriate, and very appropriate, respectively. The results of the 5-point scale survey showed that the participants believed that almost all verb phrases were appropriate or very appropriate, with an average score of 4.64. Therefore, the experimental materials guided by the probe words to elicit the corresponding verbs have high reliability and consistency. Before the formal experiment, the participants will go through a material learning and testing phase to activate the target verb in the first reaction during the formal experiment.

The experimental design included the phonological relatedness between response words and context pictures (related vs. unrelated) as within-participants and within-items variable and the verb context (single verb generation vs. verb phrase generation) as between-participants and within-items variable. A total of 108 probe-response-picture trials were used, with 96 for the experiment and 12 for warm-up. During the entire session, the order of items was pseudo-randomized for each participant with the constraint that a particular picture did not reoccur for at least five trials, and the first phoneme of target words in the consecutive trials was not same.

### Apparatus

The experiment was conducted using a WACOM Intuos A4 graphic tablet and a WACOM inking digitizing pen (Wacom) that were connected to a computer running the E-Prime Professional Software (Psychology Software Tools, Pittsburgh, PA). Participants were seated at approximately 70 cm. Stimuli were presented at the bottom of the screen in order to reduce head and eye movements between the screen and the writing surface. Writing latencies were collected as the intervals between the probe-context picture onset and initial contact of the pen on the writing surface. In single verb generation production, probe words were presented in 28-point Song font. Pictures were standardized to a size of approximately 6 × 6 cm. As in the study of Humphreys et al. (2010), the bottom of each word had a visual angle of 3 above the horizontal bottom midline of the screen; the top of each picture appeared at 2 above the bottom midline of the screen. In verb phrase generation production, green probe pictures and red context pictures were superimposed at the bottom center of the screen.

### Procedure

Participants were tested individually. They were asked to familiarize themselves with the experimental stimuli by looking at all the pictures, which were presented in reduced size on the computer screen, with the name for each picture printed underneath it. Next, they were asked to familiarize themselves with the probe-verb pairs and tested in order to correctly write down the verbs and verb phrases.

Each trial involved the following sequence: A fixation point (*) was presented in the middle of the screen for 500 ms, followed by a blank screen for 500 ms. Participants were instructed to write down: (1) verbs corresponding to the probe words while ignoring the context pictures in single verb generation task; or (2) verb phrases corresponding to the name of the green pictures as quickly (and as accurately) as possible while ignoring the context (red) pictures in verb phrase generation task. Stimuli disappeared when participants began to write on the tablet. Upon observing the participants complete their writing, the experimenter pressed a key, initiating the subsequent trial after a 1500 ms interval.

## Results

**FIGURE 1:**
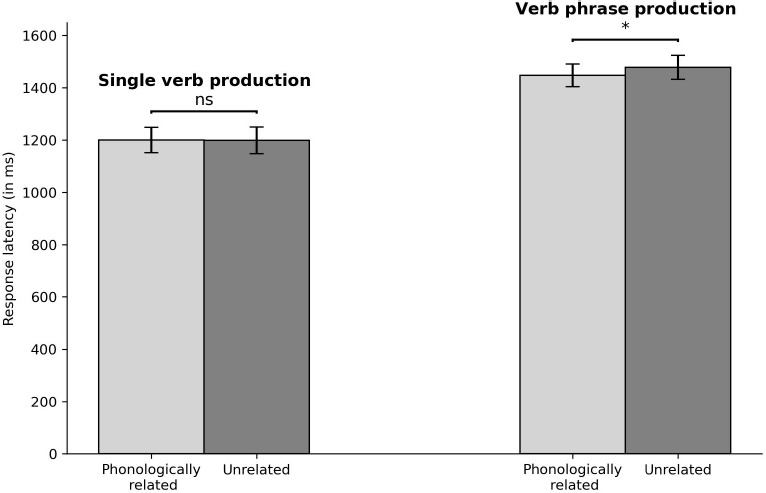
Response latency for phonologically related and unrelated conditions in single verb (LEFT) and verb phrase production (RIGHT). Error bars capture the SEM, *p < 0.05.

Data from incorrect responses (1.47%), naming latencies longer than 3,000 ms or shorter than 300 ms (0.89%), and those deviating by more than three standard deviations from a participant’s mean (1.47%) were removed from all analyses. The error rates were low and not analyzed further. Figure 1 shows the average writing latency for each condition in single verb and verb phrase generation.

**FIGURE 2:**
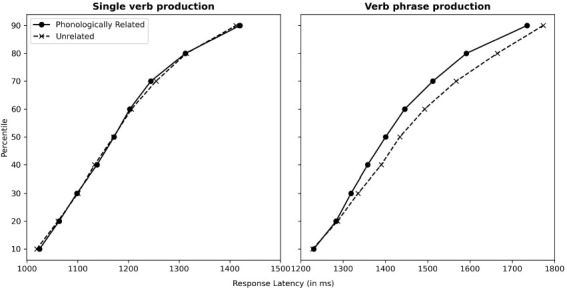
Mean cumulative response latency distributions, dependent on relatedness (phonologically related vs. unrelated) in single verb (LEFT) and verb phrase production (RIGHT).

An analysis of variance (ANOVA) was conducted on response latencies that included relatedness (phonologically related vs. unrelated) as within-participants and within-item, and verb context (single verb vs. verb phrase) as between-participants and between-item variables. The results revealed a marginally significant main effect of relatedness, *F*_1_(1, 52) = 3.67, *MSE* = 1518, *p* = 0.06; *F*_2_(1, 11) = 3.48, *MSE* = 625.11, *p* = 0.09, showing that response latencies were faster in the related condition than in the unrelated condition. A significant main effect of verb context was found, *F*_1_(1, 52) = 15.69*, MSE* = 118247.10*, p* < 0.001; *F*_2_(1, 11) = 707.41, *MSE* = 1186.38, *p* < 0.001. The interaction between relatedness and verb context was significant, *F*_1_(1, 52) = 4.40, *MSE* = 6757, *p* = 0.04; *F*_2_(1, 11) = 8.16, *MSE* = 394.19, *p* = 0.02. Tests that assessed the effects of relatedness in single verb and verb phrase generation separately showed significant facilitation (*Mdiff* = −30 ms) at verb phrase generation, *t*_1_ (29) = −2.62, *p* = 0.014; *t*_2_ (11) = −3.396, *p* = 0.006, but not in single verb generation (*Mdiff* = 1.3 ms), *ts* < 0.3, *ps* > 0.77. The cumulative frequency distributions of latencies, as depicted in Figure 2, are derived from individual calculations for each participant and decile, followed by an averaging procedure (Roelofs, 2008). This result clearly shows that the phonological effect in verb phrase production is predominantly observed across the entire latency range.

## Discussion

By eliciting verbs and verb phrases through objects, this study extended the classical word association and PPI paradigms, investigating multiple phonological activation in Chinese written production. The findings revealed an absence of multiple phonological activation in the single verb generation of written form. However, in the verb phrase generation task, a phonological relationship between distractor pictures and target verbs sped up writing latencies. Those results provide clear evidence that phonological codes constrain written word production (see Qu et al., 2011). More importantly, information transmission on semantic-to-lexical phonology in a cascaded manner.

This aspect is not easily discernible in Indo-European languages, primarily due to the shallow orthographic-phonemic consistency. Alphabetic languages typically exhibit a compounding of graphemes and phonemes, making the isolation of pure phonological effects particularly challenging, especially concerning lexical phonology. In contrast, Chinese, as a logographic language, has a unique attribute where each character corresponds to a syllable, with the syllable, rather than the phoneme, serving as the primary processing unit in Chinese speech production (O’Seaghdha et al., 2010). Recently, Wang and Zhang’s (2022) findings of early lexical phonological effects and late sublexical effects also supported the notion that activation in Chinese written production initially transfers from the semantic system to lexical phonology. The separation of phonology and orthography affords Chinese a distinctive advantage in detecting pure phonological functions within the context of written production.

A growing body of research has shown that phonology plays a pivotal role in written word production across diverse experimental tasks, such as picture-word interference, Stroop tasks, masked priming [see an overview in Qu et al. (2015)]. There remains a notable lack of consensus on how phonological representations interface with, and potentially modulate, orthographic structure. The variability in findings across different investigations underscores the complexity of this interaction and highlights the challenges in arriving at a unified understanding. A substantial body of research has focused on examining the relative temporal processes of phonology and orthography as outlined in the introduction, while overlooking the interplay between semantic and lexical phonology.

The findings of multiple phonological activation in this study are consistent with the few existing research that has delved into the interaction between semantics and phonology in written production (Bonin and Fayol, 2000). This alignment was established through a factorial design that intricately intertwined semantic and phonological relatedness, revealing a significant diminution of semantic effects under conditions of mixed relatedness, a finding that echoes the outcomes of studies centered on spoken language production in Western alphabetic languages (Starreveld and La Heij, 1995, 1996; Damian and Martin, 1999).

What are the implications of the present findings for the current theoretical frameworks of word production? To produce a target word, a central issue concerns the activation time point when different word components become available (Strijkers and Costa, 2016). The findings of multiple phonological activation in verb phrase generation underscore the principle of cascadedness in information transmission during written production.

Interestingly, an exclusive focus on single verb production could potentially lead to misleading conclusions. The absence of multiple phonological activation in this context aligns with findings from a few noun-based written (outlined in the Introduction) and spoken production in various tasks. For instance, no phonological facilitation was detected in the English-to-Chinese word translation task, even with the percentage of phonologically related trials increased to enhance sensitivity to phonological relatedness, and in the word association task, when the distractor picture name “雪人” (/xue3ren2/, snowman) was phonologically related to the target word “学生” (/xue2sheng1/, student) elicited by the probe word “老师” (/lao3shi1/, teacher) in Chinese (Zhang and Zhu, 2016). These observations underscore the complexity of the interplay between semantic and phonological processes in language production and highlight the necessity for nuanced approaches in future research endeavors to unravel the intricate dynamics underpinning these processes.

Alternatively, an augmentation in the semantic and syntactic involvement of verbs within verb phrases yields convincing evidence of multiple phonological activation. This finding underscores the intricate dynamics that characterize the processing of verbs, illuminating the profound impact of enhanced semantic and syntactic participation on the magnitude of phonological activation. Correspondingly, previous studies have confirmed that increased semantic activation aids in the detection of multiple phonological activation. For instance, multiple phonological activation was observed only when semantic activation was amplified through a semantic blocking manipulation, alongside the presentation of mediated distractor words that were phonologically related to a semantic competitor (Zhang et al., 2018), or scenarios involving synonyms (Peterson and Savoy, 1998).

A growing body of evidence indicates that differences in noun and verb processing arise primarily when words are deployed grammatically by context (Vigliocco et al., 2011). For instance, in lexical decision and semantic categorization tasks, Tyler et al. (2004) observed consistent left-lateralized activations in the inferior frontal gyrus (IFG) and temporal cortex for both nouns and verbs when presented as uninflected stems. However, the introduction of an inflectional affix elicited differential activations in the left IFG for inflected verbs compared to inflected nouns. These findings suggest that the neural differentiation between nouns and verbs is not inherent but emerges in syntactic contexts that necessitate morphological processing. The results underscore that the lexical representation in the brain does not prioritize grammatical category as a primary organizing principle. Instead, the differential processing demands of nouns and verbs, stemming from their unique roles in sentence interpretation, drive the observed neural distinctions (Tyler et al., 2001).

Nevertheless, as can be seen from introduction, early studies of brain-damaged patients showed a pronounced dissociation between noun and verb production deficits resulting from lesions in distinct cortical areas (see section “Introduction”). Utilizing event-related functional MRI, Schapiro et al. (2006) identified the specific cortical areas as English-speaking participants produced noun or verb phrases. Notably, the left prefrontal cortex and the left superior parietal lobule exhibited heightened activation during verb production compared to noun production. Conversely, the left inferior temporal lobe demonstrated increased activity during noun production. This led to hypotheses that nouns and verbs may be represented and processed in different neural networks.

Whether processed in shared or distinct cortical networks, verbs and nouns elicit different degrees of activation due to the cognitive demands of lexical semantics, which are often more complex for verbs (Yokoyama et al., 2006). The abstract and ambiguous nature of verbs is not confined to their production but is also evident in the early stages of learning, manifesting distinct challenges compared to nouns (Crossley et al., 2013). For instance, verbs are less amenable to acquisition through repetition, a technique that is often effective for nouns. The comprehension and usage of verbs are heavily contingent upon the diversity of contextual settings, underscoring their dynamic and context-dependent nature. Convergingly, it led to the heavy cognitive demands associated with lexical semantic processing when producing verbs. As pointed out by Huttenlocher and Lui (1979), unlike in object domain, action domain lacks clear hierarchical organization. Verbs impose greater difficulty than nouns and with longer latencies (Vigliocco et al., 2002). Feng et al. (2019) highlighted the complexity of verb acquisition and comprehension in comparison to nouns in Chinese. Their research, which deliberately excluded syntactic cues, emphasized the intricate semantic components inherent to verbs and their role in differentiating the neural mechanisms underlying noun and verb processing.

Chinese, characterized as a monosyllabic language, lacks the inflectional morphological changes found in Indo-European languages like English. For example, in English, verbs are inflected for tense (e.g., walk vs. walked), and nouns can be inflected for number (e.g., cat vs. cats). In Chinese, meaning words typically do not change their form through inflection. The language relies more on word order, auxiliary words, and context to convey meaning that would be expressed through inflection in other languages. It is plausible that verbs require even greater semantic and syntactic processing during lexical access relative to nouns. The increased complexity of verbs necessitates tighter linkage between lexical selection and phonological encoding to enable cascaded activation critical for retrieving their intricate forms. Results provide direct empirical evidence on Tainturier and Rapp’s (2001) interesting assumption that phonology is more heavily involved in writing sentences than in writing single words.

Based on current findings, the role of phonology, especially that originating from lexical phonology, has been significantly underestimated in the context of written production. The present study offers direct evidence on the activation of multiple phonological nodes in writing. This implies that the transmission of information from semantic to phonology within the writing system operates through a mechanism of cascading activation.

However, it is crucial to note that this cascading activation tends to manifest as a weak activation. It necessitates sensitive experimental paradigms and is contingent upon specific experimental tasks. Correspondingly, the existing evidence suggests that cascading is not “universal” such that all activated units at higher level necessary transmit activation to lower levels. For example, Kuipers and La Heij (2009), supported by Dumay and Damian (2011), introduced the concept of “limited cascadedness.” This principle posits that certain properties tied to the core identity of a target dimension, like an object’s name, are allowed to cascade down to the form level. In contrast, modifying attributes, such as color or size, are restricted from this cascading effect. The extent and nature of cascadedness are not static but can be influenced by various external factors, including the level of attention and the specific demands of a task, as highlighted by Mädebach et al. (2011). This nuanced perspective underscores the selective and conditional nature of the cascading process in cognitive processing.

Indeed, there is a same concern on the potential mis-selection due to the superimposed cue pictures and context pictures (Roelofs, 2008), considering the fact that the way of stimuli presentation in the verb phrase generation task, is identical to typical PPI, with the difference of generating verb phrases rather than picture names only. Roelofs (2008) ruled out the mis-selection theory by analyzing response latency distributions in related and unrelated conditions. The effect was observed consistently across all latency ranges and increased linearly with latency instead of showing effect in slow responses only. Here a similar result was found that the effect was consistently across almost all latency ranges, supporting multiple phonological activation in Chinese verb written production (Figure 2).

In conclusion, to my knowledge, this study provides initial evidence for multiple phonological activation during Chinese written verb production. This signifies phonology, especially lexical phonology, plays a larger role in writing in Chinese, a logographic language, than previously assumed. Alongside direct semantic-to-orthography mapping, these findings elucidate the nature of information transmission between semantic and lexical phonology depicted in writing production models (Bonin et al., 2001). Moreover, due to the paucity of research on cascaded phonological activation in writing, current results cannot be directly compared to findings from speech production, which increasingly suggest writing does not merely follow speech but possesses its own characteristics. For now, combined with Qu and Damian’s (2015) evidence for cascaded semantic-to-orthography activation, a convincing conclusion can be drawn: In written production, activation firstly transmits semantic information in a non-serial, parallel fashion to both lexical phonology and orthographic levels, consistent with a weak cascade activation pattern.

## Data availability statement

The raw data supporting the conclusions of this article will be made available by the authors, without undue reservation.

## Ethics statement

The studies involving humans were approved by the Independent Ethics Committee of Shanghai International Studies University. The studies were conducted in accordance with the local legislation and institutional requirements. The participants provided their written informed consent to participate in this study.

## Author contributions

XZ: Writing – original draft.
